# The outcome of digital technology in microvascular free flap reconstruction for ORNJ: a retrospective study

**DOI:** 10.3389/fbioe.2026.1842912

**Published:** 2026-06-01

**Authors:** Xinyue Xu, Zihao Tang, Dandan Wang, Chenlu Li, Jihong Wang, Yiwen Liu, Lei Tian, Chunlin Zong

**Affiliations:** National Clinical Research Center for Oral Diseases, State Key Laboratory of Oral and Maxillofacial Reconstruction and Regeneration, Shaanxi Clinical Research Center for Oral Diseases, Department of Oral and Maxillofacial Surgery, School of Stomatology, The Fourth Military Medical University, Xi’an, Shaanxi, China

**Keywords:** craniofacial reconstruction, digital workflow, microvascular free flap reconstruction, osteoradionecrosis of the jaw, three-dimensional printing, virtual surgical planning

## Abstract

**Background:**

Osteoradionecrosis of the jaw (ORNJ) is a serious and refractory late complication of radiotherapy used in the treatment of head and neck cancer. Microvascular free flap reconstruction is an ideal option to reconstruct the residual defect after surgical resection of ORNJ lesion. The application of virtual surgical planning (VSP) and three-dimensional printing (3DP) can effectively improve the reconstructive results with free flaps in jaw bone defect. However, the effect of VSP and 3DP in ORNJ reconstruction with free flaps is still unclear. A retrospective study was conducted to evaluate the free flap types, the success rate, and complications in ORNJ.

**Methods:**

The ORNJ inpatients that underwent free flaps reconstruction in our hospital between 2012 and 2024 were included in the study and divided into the conventional flap (CF) and digital flap (DF) groups with the application of VSP and 3DP. The patient characteristics and microvascular free flap reconstruction outcome were reviewed.

**Results:**

A total of 35 ORNJ patients with 39 free flaps were included in the study. The success rate of free flaps reconstruction in ORNJ was 89.7%, with the postoperative early complication rate of 58.9% and late complication rate of 25.6%. Fibula was the most commonly used flap type (76.9%) of ORNJ defect reconstruction and had the lowest complication rate (63.3%). The success rate between the CF (92.9%) and DF (88%) groups had no significant difference. The mean surgical duration and the length of hospital stay in the DF group were significantly shorter than those of the CF group. Moreover, the difference of the postoperative complication rate between the CF (78.6%) and DF (64%) groups was not statistically significant. The fistula formation rate was significantly lower in the DF group than in the CF group.

**Conclusion:**

Microvascular free flap reconstruction is safe and effective for advanced osteoradionecrosis of the jaw, with the fibula flap representing the most favorable donor site. The adjunctive use of virtual surgical planning and three-dimensional printing significantly reduces the operative time, length of hospital stay, and fistula formation. These digital techniques are valuable adjuvant tools that optimize surgical efficiency and decrease critical complications in ORNJ reconstruction.

## 1 Introduction

Osteoradionecrosis of the jaw (ORNJ) is a serious and refractory late complication of radiotherapy used in the treatment of head and neck cancer, with an incidence of 2%–22% (Ohori et al., 2024; Teng and Neal, 2005). ORNJ is defined as exposed necrotic bone after radiation, which does not show signs of healing over 3 months, without evidence of persisting or recurrent cancer (Chronopoulos et al., 2018; Havndrup-Pedersen et al., 2025). The clinical presentation of ORNJ includes exposed necrotic bone, recurrent infection, swelling, pain, orocutaneous fistulas, trismus, xerostomia, dysphagia, and even pathologic fracture, which adversely impact the quality of life of the patients (Havndrup-Pedersen et al., 2025). Unfortunately, the pathogenesis is still not fully understood, combined with no universally accepted gold standard treatment protocol for ORNJ.

The therapeutic paradigm of ORNJ includes conservative treatment and surgical treatment. Conservative measures (e.g., oral hygiene, antibiotics, hyperbaric oxygen, and sequestrectomy) may alleviate symptoms but rarely halt disease progression (Md et al., 2021). Therefore, surgical resection of the necrotic bone and the surrounding affected tissues is essential for refractory or advanced ORNJ. The residual extensive tissue defects after surgery, including that in jaw bone and soft tissue, should be reconstructed to avoid facial deformity and oral dysfunction.

Despite widespread use, free flap reconstruction in ORNJ patients remains challenging. Radiation-induced tissue fibrosis, vascular injury, and persistent infection contribute to relatively high complication and flap failure rates (Kostares et al., 2024; Arianpour et al., 2024). The reported flap failure rates range from 3.1% to 16%, with overall complication rates between 8% and 43% (Farsi et al., 2025; Eckardt and Fokas, 2003; Kostares et al., 2024; Lee et al., 2015). In recent years, digital technologies including virtual surgical planning (VSP) and three-dimensional printing (3DP) have been increasingly adopted in head and neck reconstruction to improve surgical accuracy and efficiency (Lee et al., 2025). Preliminary studies indicated that VSP/3DP may enhance the precision and outcomes of free flap reconstruction in ORNJ (Huang et al., 2023; Annino et al., 2022; Chen et al., 2018). VSP primarily involves determining the osteotomy extent for necrotic bone resection, along with planning the length and contouring of the free flap (Tang et al., 2019; Jelovac et al., 2023). 3DP is mainly used to manufacture patient-specific osteotomy guides, positioning guides, and a stereolithic model of the mandible, which allows for pre-bending of the reconstruction plate preoperatively (Rodríguez-Arias et al., 2024). However, high-quality evidence comparing digital-assisted surgery with conventional freehand techniques remains limited.

To analyze the outcome of digital technology in the reconstruction for ORNJ with microvascular free flaps, the present single-center retrospective study was performed to compare conventional freehand surgery *versus* VSP/3DP-assisted surgery in patients undergoing free flap reconstruction for ORNJ. The flap selection, flap survival, early and late complications, surgical duration, intraoperative blood loss, length of hospital stay, hospital costs, and specific complication patterns were systematically evaluated.

## 2 Patients and methods

### 2.1 Patients

This study was conducted following approval by the Institutional Review Board of the School of Stomatology, the Fourth Military Medical University, and adhered to the tenets of the Declaration of Helsinki.

The study utilized a retrospective cohort design, including ORNJ patients treated at the Department of Oral and Maxillofacial Surgery, School of Stomatology, Fourth Military Medical University, from January 2012 to December 2024. The inclusion criteria were limited to ORNJ patients who underwent simultaneous resection and microvascular free flaps reconstruction surgery. There were no restrictions on age, sex, or comorbidities. Patients with incomplete clinical records were excluded. In addition, patients who did not undergo microvascular free flap reconstruction, had a follow-up duration of less than 1 year, underwent staged resection and reconstruction, or had received vascularized flap reconstruction before radiotherapy were also excluded (Ho et al., 2016). The choice between conventional freehand and digital-assisted reconstruction was based on the clinical availability of digital planning technology, the patients’ willingness, and the surgeon’s preference. A flowchart detailing the inclusion and exclusion criteria is presented in Supplementary Figure S1. No strict randomization was applied, which is consistent with real-world clinical practice. In this retrospective study, patients were consecutively enrolled, and the numerical discrepancy in the sample size between the conventional flap (CF) and digital flap (DF) groups reflected real-world clinical practice rather than selective allocation. Baseline characteristics were comparable between the two groups (Supplementary Table 1). For each patient, the general information, primary cancer, teeth extraction history after radiotherapy, total radiotherapy dose, the time between irradiation and the diagnosis of ORNJ, the time between diagnosis and reconstruction surgery, free flap types, and complications were recorded. Patients were followed up postoperatively through outpatient clinic visit for at least 1 year. CT scans were taken at 1 week and 6 months to 1 year after surgery. Success of free flap transplantation and reconstruction was defined as complete survival and uncomplicated healing of the original flap for more than 1 year after surgery, whereas flap failure was defined as any condition requiring flap resection due to various complications. Complications were defined as any adverse event within 30 days (early) or after 30 days (late) postoperatively, including infection, fistula, wound dehiscence, and vascular thrombosis.

Based on the application or absence of VSP and 3DP technique, the patients were divided into the DF and CF groups. In the DF group, patients underwent jaw bone resection and reconstruction utilizing VSP and 3DP templates, whereas patients in CF group were treated with freehand surgery. The free flap success rate, complication rate, surgical duration, intraoperative blood loss, length of hospital admission, and mean hospital charge were compared between two groups.

### 2.2 Operative procedures

**FIGURE 1:**
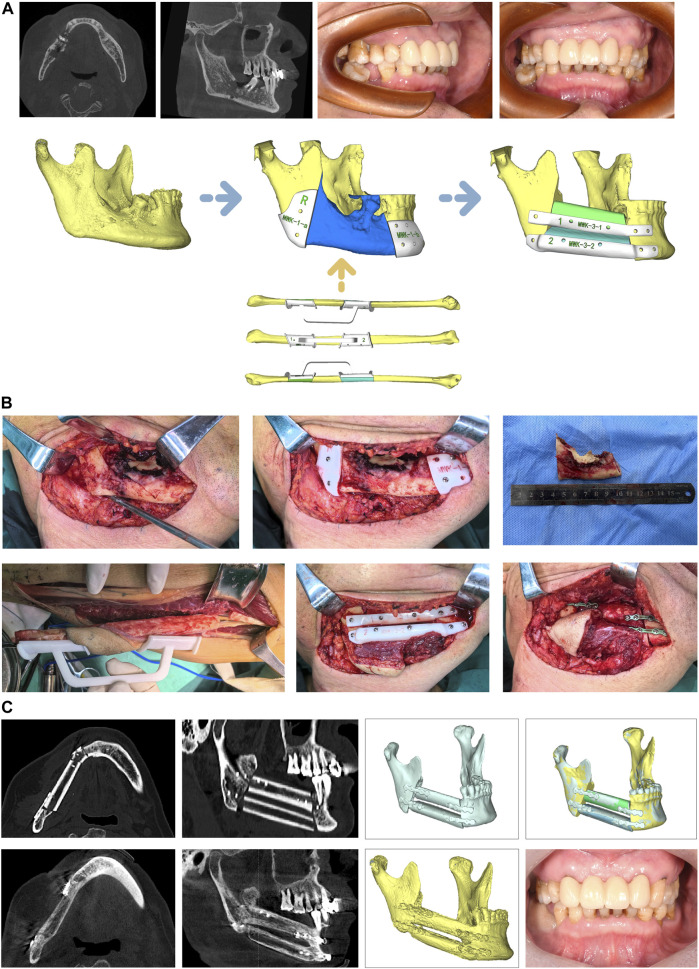
Digital workflow of free flaps reconstruction for ORNJ. **(A)** Preoperative analysis of ORNJ lesion and virtual surgical planning of the surgical resection range, reconstruction method with the fibula flap, and design of the guiding templates. **(B)** Intraoperative application of the 3D printed templates. **(C)** Postoperative results.

Before surgery, all patients underwent head and neck CT scan to evaluate the necrotic bone lesion, and lower extremity CT angiography was scanned if it was necessary. In the DF group, the CT images in the DICOM format were imported into a virtual surgical planning software application (Mimics, version 19.0, Materialise, Leuven, Belgium). Total resection of the necrotic and unhealthy jaw bone was planned in the software application, with at least a 1-cm safety margin. If the defect were planned to be reconstructed by bony flaps, such as the fibula and iliac crest, the resection and cut margins with the localization of the optimal angles for performing osteotomies was also designed by the software application. Then, the surgical guides were designed and printed with a professional 3D printer (J750, Stratasys, Eden Prairie, MN, USA). In addition, the mandibular or maxillary model was printed to pre-bend the titanium plates. During surgery, the neck vasculature was dissected and evaluated, and then the jaw bone was resected with the guidance of printed osteotomy templates. The donor bone was also harvested, contoured to fit the jaw bone defect, and placed onto the defect site by the osteotomy templates according to the virtual surgical plan. With the help of the digital technique, the resection of necrotic jaw bone and the harvesting of donor bone can be performed at the same time. Then, the shaped donor bone was fixed to the jaw bone using pre-bent titanium plates, followed by vascular anastomosis. The procedure of VSP- and 3DP-assisted resection of ORNJ lesion and reconstruction with a fibula flap is shown in Figure 1. In the CF group, no pre-bent plates or surgically guided templates were used during surgery, which was performed mainly based on the surgeon’s experience. After the dissection of the neck vascular, the necrotic jaw bone was totally resected, and the length of defect was measured using a ruler to guide the donor bone harvesting. Then, the suitable length of donor bone was harvested, shaped to fit the defect, and fixed to the jaw bone depending on the surgeon’s experience.

### 2.3 Data analyses

The statistical analysis was performed using SPSS statistical software. The continuous quantitative variables were described as the mean ± SD for normally distributed data and median (interquartile range, IQR) for non-normally distributed data, and the qualitative variables were presented as frequencies (N) and relative distributions (%). The normally distributed continuous data were compared by Student’s t-test, and Mann–Whitney test was utilized for the non-parametric data. The categorical data were compared using the chi-square test. Statistical significance was considered as *P* < 0.05.

## 3 Results

### 3.1 Patients characteristics

From 2012 to 2024, there were 186 cases comprising 131 ORNJ inpatients (some patients were hospitalized more than once) who underwent surgeries in our hospital. Patients averaged 57.1 ± 12.2 years old, and 31.8% of the patients were female. Among these, 35 ORNJ patients who underwent reconstructive surgery using free flaps were included, and 39 free flaps were conducted in total. The free flap reconstruction rate of ORNJ was 26.7%. The average age of the patients was 55.9 ± 11.7 years, with 10 (28.6%) female patients and 25 (71.4%) male patients. The primary tumor serving as an indication for radiotherapy in the head and neck was maxillofacial squamous cell carcinoma (16 patients, 45.7%), nasopharyngeal carcinoma (nine patients, 25.7%), adenoid cystic carcinoma (four patients, 11.4%), lymphoma (three patients, 8.6%), malignant pleomorphic adenoma (two patients, 5.7%), and myxofibrosarcoma (one patient, 2.9%). The mean radiation dose was 61.8 Gy, and 34.3% patients had a history of teeth extraction after radiotherapy. Most of the flaps were used in the mandible (37 cases, 94.9%). Patient characteristics are shown in Supplementary Table S1.

### 3.2 Microvascular free flap reconstruction outcomes

The flap types of ORNJ defect reconstruction included the fibula (30, 76.9%), radial forearm (4, 10.3%), iliac crest (3, 7.7%), and anterolateral thigh (2, 5.1%) flaps. The success rate of free flaps reconstruction in ORNJ was 89.7% (35/39). The success rate of different flaps was 93.3% (28/30) for fibular flap, 66.7% (2/3) for iliac crest flap, 75% (3/4) for radial forearm flap, and 100% (2/2) for anterior-lateral thigh flap, respectively. In total, four flaps failed (10.3%), including two fibula, one iliac crest, and one radial forearm flaps, and the failures were rescued by pectoralis major myocutaneous flaps. Of those that failed, one had infection, two had vessel thromboses, and one had blood leakage at the vascular anastomosis site and infection. The postoperative early complication (<30 d) rate was 58.9%, and the late complication (>30 d) rate was 25.6%. Among the different flap types, the complication rate was the lowest in the fibula flap (63.3%). The reconstruction results of different flaps in ORNJ are shown in Table 1.

**TABLE 1:** Reconstruction outcome of different flaps.

Flap type	Reconstruction rate (n, %)	Success rate (n, %)	Complication rate (n, %)
Fibula	30, 76.9%	28, 93.3%	19, 63.3%
Radial forearm	4, 10.3%	3, 75%	3, 75%
Iliac crest	3, 7.7%	2, 66.7%	2, 66.7%
Anterolateral thigh	2, 5.1%	2, 100%	2, 100%

### 3.3 The surgical outcomes of digital-assisted reconstruction

Both the DF and the CF groups utilized fibula flaps, iliac crest flaps, radial forearm flaps, and pectoralis major flaps. There were 14 flaps in the CF group and 25 flaps in the DF group. The mean age was 52.1 ± 13.2 years in the CF group and 56.9 ± 13.2 years in the DF group. The gender included 10 males and four females in CF group and 18 males and seven females in the DF group. The defect size was 11.0 ± 4.5 cm and 12.0 ± 3.2 cm in the CF and DF groups, respectively. There were no statistically significant differences in these baseline characteristics between the two groups, as shown in Table 2.

**TABLE 2:** Baseline characteristics in the CF and DF groups.

Variable	CF group (n = 14)	DF group (n = 25)	*P-*value
Age	52.1 ± 13.2	56.9 ± 13.2	0.277
Sex			
Male	10	18	​
Female	4	7	0.970
Defect Size (cm)	11.0 ± 4.5	12.0 ± 3.2	0.444

Of the 14 flaps in the CF group, 13 (92.9%) achieved successful reconstruction, while the DF group had 22 of 25 flaps (88%) with successful reconstruction. The success rate between two groups had no significant difference (*p* = 0.608). The mean surgical duration in the DF group (392.2 ± 67.0 min) was found to be significantly shorter (*p* = 0.021) than that in the CF group (464.0 ± 90.5 min). The intraoperative blood loss was 532.1 ± 250.1 mL in the CF group and 408 ± 108.7 mL in the DF group, and there was no statistically significant difference (*p* = 0.096). The length of hospital stay in the CF group (42.2 ± 20.1 nights) was significantly longer (*p* = 0.033) than that in the DF group (29.6 ± 15.1 nights). The mean hospital charge was more expensive in the DF group (95,600 ± 16,800 RMB) than in the CF group (58,200 ± 28,500 RMB). The results of these variables in the two groups are shown in Table 3.

**TABLE 3:** Reconstructive results in the CF and DF groups.

Parameters	CF group (n = 14)	DF group (n = 25)	*P-*value
Success rate	92.9%	88%	0.608
Surgical duration (minutes)	464.0 ± 90.5	392.2 ± 67.0	0.021
Blood loss (mL)	532.1 ± 250.1	408 ± 108.7	0.096
Length of hospital stay (nights)	42.2 ± 20.1	29.6 ± 15.1	0.033
Hospital charge (RMB)	58,200 ± 28,500	95,600 ± 16,800	0.032

Moreover, the complication rate between the two groups was also analyzed. In the CF group, 11 (78.57%) patients had postoperative complications, and 16 (64%) patients had postoperative complications in the DF group. However, the difference between the two groups was not significant (*p* = 0.321). Furthermore, the specific complication type was also studied. As shown in Table 4, the fistula formation rate was significantly lower in the DF group than in the CF group, while other complications had no statistically significant difference between the two groups.

**TABLE 4:** Postoperative complications in the CF and DF groups.

Complications	CF group (n = 14)	DF group (n = 25)	*P-*value
Postoperative infection	6, 35.7%	14, 60%	0.244
Fistula formation	4, 28.6%	0, 0%	0.018
Skin paddle necrosis	1, 7.1%	3, 12%	1.000
Non-union	2, 14.3%	0, 0%	0.114
Wound dehiscence	2, 14.3%	5, 20%	1.000
Vascular thrombosis	1, 7.1%	3, 12%	1.000

## 4 Discussion

As a late complication of radiotherapy in the head and neck region, the treatment of ORNJ is a challenging issue. In advanced cases, large necrotic bone is formed, in which conservative treatment is not sufficient and surgical resection is indicated (Costantino et al., 2025). Microvascular free flap reconstruction is an ideal option to reconstruct the residual defect after surgical resection of ORNJ lesion. Unfortunately, the free flap reconstruction in ORNJ is difficult, and further analysis is needed to evaluate the treatment effect. The application of VSP and 3DP can effectively improve the reconstructive results with free flaps in jaw bone defect (Ma et al., 2021). However, the effect of these digital technics in ORNJ reconstruction with free flaps is still unclear. In our study, we found that 26.7% of ORNJ inpatients received free flap reconstruction, and the success rate of free flaps reconstruction in ORNJ was 89.7%, with a high postoperative early complication rate of 58.9%. The application of VSP and 3DP did not improve the success rate or reduce the complication rate significantly, but it significantly shortened the surgical duration, reduced the length of hospital stay, and decreased the fistula formation rate.

In our study, we found that ORNJ patients averaged 57.1 ± 12.2 years old and were predominantly male (68.2%), which was similar to that in published literature. A meta-analysis study (Farsi et al., 2025) including 397 ORNJ patients showed that patients averaged 54.8 years old, and 27.5% were female. Consistent with other studies, we also found squamous cell carcinoma and nasopharyngeal carcinoma was the main reason for radiotherapy in ORNJ patients (Farsi et al., 2025; Li et al., 2020). Although teeth extraction was considered a high risk of ORNJ (Topkan et al., 2023), only 34.3% of patients had a history of teeth extraction after radiotherapy in our study, which may be attributed to the high radiation dose in these patients. We also found that most of the flaps were used in the mandible (94.9%), which was consistent with the reports of other studies (Farsi et al., 2025; Baumann et al., 2011). The incidence of ORNJ in the mandible is higher than that in the maxilla (Frankart et al., 2021), and a segmental resection of ORNJ lesion in the mandible can be better reconstructed with free flaps than other methods, such as prosthetics (Haroun and Coblens, 2019).

The donor-site of free flaps varied in reconstructive surgery of jaw bone defect. In our study, we also found that 76.9% of the ORNJ was reconstructed by a fibula flap. The advantages of the fibula include its thick cortical bone, long harvested distance, suitable vessel diameters that closely match the recipient vessels in the head and neck, and long vascular pedicle, among others. In our study, the scapula flap was not used, while it was used in literature for ORNJ reconstruction (O'Connell et al., 2021; Bettoni et al., 2019). The advantage of the scapula flap is the abundant soft-tissue and two discrete skin paddles that can make it a chimeric flap. However, the drawback includes its limited length, thin bone, and the need for patient repositioning during the surgery for harvest. The failure rate of free flaps reconstruction in ORNJ was 10.3%, which correlates with other studies, where it ranged from 5% to 16% (O'Connell et al., 2021; Lee et al., 2015; Baumann et al., 2011). In our study, the postoperative early complication rate was 58.9%, and the late complication rate was 25.6%, which are higher than the previously reported ranges (Celik et al., 2002; Coskunfirat et al., 2005). Moreover, we found that the fibular flap had the highest success rate and the lowest complication rate, which was consist with most studies (Farsi et al., 2025).

For the free flap reconstruction surgery of ORNJ, several case reports showed that VSP and osteotomy templates could maximize treatment efficacy and achieve functional and esthetic results (Chen et al., 2018; Huang et al., 2022). A retrospective study analyzed the accuracy of bony free flap reconstruction with VSP and 3D-printed-guide for mandibular ORNJ (Annino et al., 2022). The researchers found that VSP and 3D-printed-guide aided ORNJ reconstruction with accurate results, acceptable complications, and improved function. However, few studies compare the treatment results of VSP and 3D-printed-guide with conventional free-hand procedures in the surgery of ORNJ reconstruction with free flaps. In our study, we found that VSP and 3D-printed-guide could not improve the success rate of ORNJ reconstruction with free flaps. The failure of free flaps in ORNJ reconstruction was mainly attributed to the fibrotic soft tissue bed, vascular damage by radiation, microorganisms, and the complex defect environment (Kostares et al., 2024; Arianpour et al., 2024), which cannot be overcome by VSP and 3D-printed-guide. In our study, the VSP and 3D-printed-guide could significantly shorten the surgical duration and reduce the length of hospital stay. In a retrospective study, the authors compared the computer-assisted surgery (CAS) and traditional freehand surgery in mandibular reconstruction with a fibular flap (Ma et al., 2021). They found that the average operation time and hospitalization were less, which was similar to the findings in our study. They also found that CAS reduced the bleeding volume, but our results showed that VSP and 3D-printed-guide did not reduce the intraoperative blood loss.

Digital-assisted techniques have been increasingly applied in flap reconstruction surgery, primarily aiming to improve surgical efficiency, accuracy, and safety (Yu et al., 2026; Rodríguez-Arias et al., 2024). However, the present study did not demonstrate a statistically significant improvement in the flap survival rate. Although the success rate in the digital-assisted (DF) group (92.9%) was higher than that in the conventional (CF) group (88.0%), the difference was not statistically significant. This finding indicates that factors determining flap survival may not be primarily influenced by digital techniques. Instead, the key determinants include thorough debridement of necrotic bone, adequate vascular supply, and effective postoperative infection control. Previous studies have shown that the presence of non-viable bone margins increases the recurrence rate by 11.9%, highlighting the importance of radical resection (Doub et al., 2024). Moreover, in patients with osteoradionecrosis (ORN), impaired healing capacity remains the fundamental challenge. Radiation-induced damage leads to a hypoxic, hypocellular, and hypovascular tissue environment, which severely compromises wound healing regardless of surgical precision (Lin et al., 2023). In addition, conventional surgical approaches are already well-established with relatively high success rates, leaving limited room for further improvement. It is also notable that the routine use of vascularized flaps itself substantially enhances surgical success and reduces complications, potentially overshadowing the incremental benefits of digital assistance. However, digital techniques may still provide significant advantages in unity with limited surgical experience by standardizing the procedures and improving reproducibility.

In contrast, digital-assisted surgery demonstrated a clear advantage in reducing the incidence of postoperative fistula formation. The development of fistula after surgery for ORNJ is multifactorial. Radiation-induced tissue damage leads to a hypoxic, hypocellular, and hypovascular environment, which predisposes patients to poor healing and infection (Lin et al., 2023). In addition, residual non-viable tissue at the surgical margin can lead to infection recurrence and subsequent fistula formation (Doub et al., 2024). Pre-existing cutaneous fistulas are also strong risk factors for postoperative complications. Furthermore, high-dose radiotherapy (≥80 Gy) and bilateral ORN involvement have been associated with an increased risk of postoperative infection and fistula development. Mechanical factors, such as titanium plate implantation, wound dehiscence, and partial flap necrosis, also contribute to fistula formation.

Digital-assisted techniques may reduce fistula incidence through several mechanisms. First, preoperative planning based on imaging and three-dimensional reconstruction can significantly shorten the operative time, thereby reducing intraoperative exposure and the risk of infection (Neto et al., 2016). Second, these techniques enhance the morphological accuracy of osteotomy and reconstruction, enabling precise matching between the defect and the grafted flap (Bardol et al., 2020). This precise fit reduces excessive intraoperative soft tissue dissection and manipulation, thereby minimizing tissue trauma, ischemia, and subsequent inflammatory responses. Third, improved surgical precision allows for complete resection of necrotic bone and tissue while minimizing damage to adjacent healthy tissue (Bardol et al., 2020; Pabst et al., 2026). This accurate debridement and optimal flap adaptation effectively eliminate dead space, thereby reducing the risks of fluid accumulation, infection, and other postoperative complications. Precise plate design minimizes soft-tissue irritation and mechanical stress, thus reducing the likelihood of wound breakdown (Oh et al., 2022). Fourth, virtual planning preserves perforator vessels and optimizes flap vascular supply by carefully designing the osteotomy to avoid critical vessels, thus ensuring adequate blood perfusion, promoting flap survival, and reducing the risk of flap necrosis. Collectively, these advantages contribute to improved wound healing conditions and a reduced risk of fistula formation, even though they may not significantly affect the overall flap survival.

The present study has several limitations. First, the sample size was relatively small, and the retrospective design precluded randomization, limiting the ability to establish causal relationships. Second, the cost of digital planning and three-dimensional printing technology was relatively high, which may restrict the widespread applicability of this approach in routine clinical practice. Third, the single-center design and the lack of external validation may limit the generalizability of the findings. Future prospective multi-center studies with larger sample sizes and standardized protocols are necessary to confirm our results.

## 5 Conclusion

Microvascular free flap reconstruction is safe and effective for advanced ORNJ, with the fibula flap being the preferred donor site. The use of VSP and 3DP does not improve the flap survival or reduce the overall complications, but it significantly shortens the operative time, reduces the length of hospital stay, and decreases fistula formation. These digital technologies serve as valuable adjuvant tools to enhance surgical efficiency and reduce specific complications in ORNJ reconstruction. Future prospective multi-institutional studies are necessary to validate these findings.

## Data Availability

The original contributions presented in the study are included in the article/Supplementary Material; further inquiries can be directed to the corresponding authors.
