# Supplementary material for: The outcome of digital technology in microvascular free flap reconstruction for ORNJ: a retrospective study
Source: Front Bioeng Biotechnol. 2026 Jun 1;14:1842912. doi: 10.3389/fbioe.2026.1842912 (PMC13265449; doi:10.3389/fbioe.2026.1842912)
Supplement: Supplementary file 1 [file DataSheet1.zip › Supplementary Figure 1 图注.docx]

**Supplementary Figure 1** Flowchart of patient inclusion and exclusion criteria. A total of 131 ORNJ inpatients (186 surgical procedures) from January 2012 to December 2024 were initially screened. After excluding patients who did not undergo free flap reconstruction or had incomplete clinical records, 35 patients with 39 free flaps were included in the final analysis. These were divided into the conventional freehand group (CF, n=14 flaps) and the digital-assisted group (DF, n=25 flaps). ORNJ: osteoradionecrosis of the jaw.
