# Supplementary material for: The outcome of digital technology in microvascular free flap reconstruction for ORNJ: a retrospective study
Source: Front Bioeng Biotechnol. 2026 Jun 1;14:1842912. doi: 10.3389/fbioe.2026.1842912 (PMC13265449; doi:10.3389/fbioe.2026.1842912)
Supplement: Supplementary file 1 [file DataSheet1.zip › Supplementary_Table_1.docx]

Supplementary Table 1 Patient characteristics

| **ID** | **Age** | **Gender** | **Primary tumor** | **Radiation dose** | **Teeth extraction** | **ORNJ site** |
| --- | --- | --- | --- | --- | --- | --- |
| 1 | 55 | F | Nasopharyngeal carcinoma | 56 | Yes | Mandible |
| 2 | 60 | M | Malignant  pleomorphic adenoma | 60 | Yes | Mandible |
| 3 | 63 | M | Squamous cell carcinoma | 63 | No | Mandible |
| 4 | 51 | M | Squamous cell carcinoma | 64 | No | Mandible |
| 5 | 36 | F | Lymphoma | 62 | No | Maxilla |
| 6 | 61 | F | Nasopharyngeal carcinoma | 50 | No | Mandible |
| 7 | 59 | M | Nasopharyngeal carcinoma | 70 | No | Mandible |
| 8 | 31 | F | Myxofibrosarcoma | 72 | No | Mandible |
| 9 | 66 | M | Nasopharyngeal carcinoma | 60 | Yes | Mandible |
| 10 | 56 | M | Nasopharyngeal carcinoma | 70 | No | Mandible |
| 11 | 46 | M | Squamous cell carcinoma | 65 | No | Mandible |
| 12 | 58 | M | Squamous cell carcinoma | 67 | Yes | Mandible |
| 13 | 64 | F | Squamous cell carcinoma | 66 | Yes | Mandible |
| 14 | 55 | F | Nasopharyngeal carcinoma | 72 | No | Mandible |
| 15 | 67 | F | Nasopharyngeal carcinoma | 30 | Yes | Mandible |
| 16 | 61 | M | Squamous cell carcinoma | 56 | Yes | Mandible |
| 17 | 64 | M | Squamous cell carcinoma | 60 | Yes | Mandible |
| 18 | 60 | M | Nasopharyngeal carcinoma | 60 | No | Mandible |
| 19 | 60 | M | Squamous cell carcinoma | 60 | No | Mandible |
| 20 | 31 | M | Lymphoma | 60 | No | Maxilla |
| 21 | 69 | F | Adenoid cystic carcinoma | 62 | No | Mandible |
| 22 | 43 | M | Squamous cell carcinoma | 60 | No | Mandible |
| 23 | 61 | M | Squamous cell carcinoma | 90 | No | Mandible |
| 24 | 72 | M | Nasopharyngeal carcinoma | 62 | Yes | Mandible |
| 24 | 52 | M | Adenoid cystic carcinoma | 50 | No | Mandible |
| 26 | 68 | F | Lymphoma | 60 | No | Mandible |
| 27 | 26 | M | Squamous cell carcinoma | 66 | Yes | Mandible |
| 28 | 48 | M | Adenoid cystic carcinoma | 56 | Yes | Mandible |
| 29 | 74 | M | Squamous cell carcinoma | 60 | No | Mandible |
| 30 | 66 | M | Squamous cell carcinoma | 66 | Yes | Mandible |
| 31 | 60 | M | Squamous cell carcinoma | 60 | No | Mandible |
| 32 | 58 | M | Squamous cell carcinoma | 60 | No | Mandible |
| 33 | 42 | M | Adenoid cystic carcinoma | 62 | No | Mandible |
| 34 | 56 | M | Malignant pleomorphic adenoma | 66 | No | Mandible |
| 35 | 57 | F | Squamous cell carcinoma | 60 | No | Mandible |
