# Supplementary figures and images for: The outcome of digital technology in microvascular free flap reconstruction for ORNJ: a retrospective study
Source: Front Bioeng Biotechnol. 2026 Jun 1;14:1842912. doi: 10.3389/fbioe.2026.1842912 (PMC13265449; doi:10.3389/fbioe.2026.1842912)

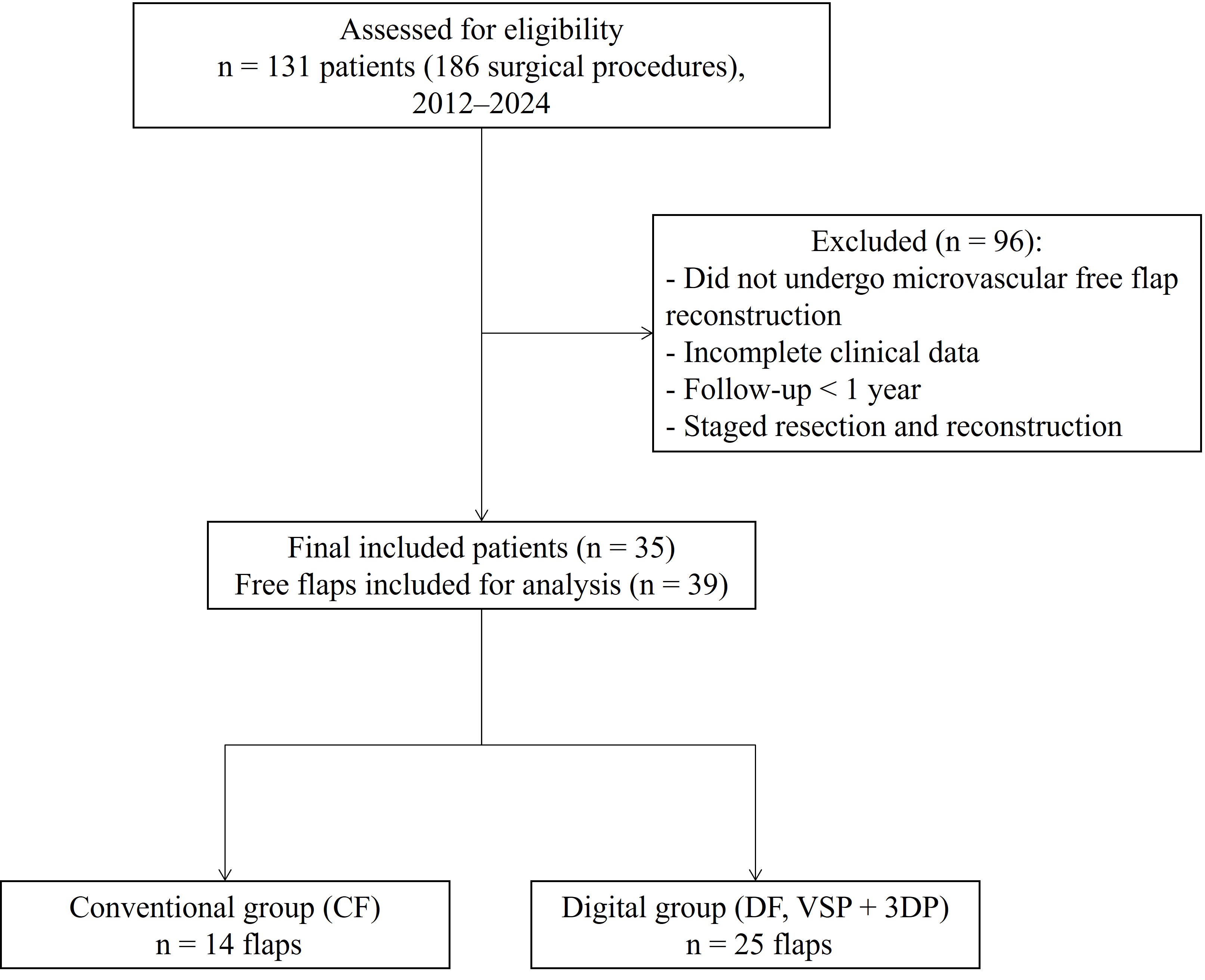

Supplement: Supplementary file 1 [file DataSheet1.zip › Supplementary Figure1.jpg]
